# Supplementary material for: Ultra-low threshold continuous-wave quantum dot mini-BIC lasers
Source: Light Sci Appl. 2023 Apr 25;12:100. doi: 10.1038/s41377-023-01130-5 (PMC10130040; doi:10.1038/s41377-023-01130-5)
Supplement: Supplementary file 1 — Supplementary Information [file 41377_2023_1130_MOESM1_ESM.pdf]

Supplementary Information for Ultra-low Threshold Continuous-wave Quantum Dot Mini-BIC Lasers

Hancheng Zhong[1], Ying Yu[1]*, Ziyang Zheng[1], Zhengqing Ding[1], Xuebo Zhao[1], Jiawei Yang[1], Yuming Wei[2], Yingxin Chen[1], Siyuan Yu[1]*

[1] State Key Laboratory of Optoelectronic Materials and Technologies, School of Electronics and Information Technology, Sun Yat-Sen University, Guangzhou 510006, China
[2] School of Physics, Sun Yat-Sen University, Guangzhou 510275, China

Corresponding author: *yuying26@mail.sysu.edu.cn; yusy@mail.sysu.edu.cn*

Section I. Epitaxial structure and material characterization.

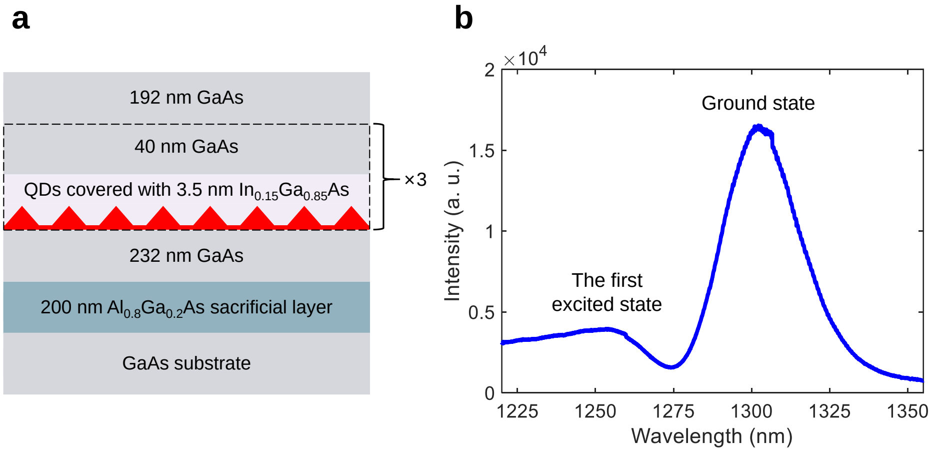

Figure S1. a. Schematic epitaxial structure for the mini-BIC laser. **b.** The photoluminescence (PL) under 1 mW pumping power at room temperature. Ground state emission peaking at 1300 nm with a narrow full-width at half-maximum (FWHM) of 30 meV was observed, together with the first excited state at ~1255 nm.

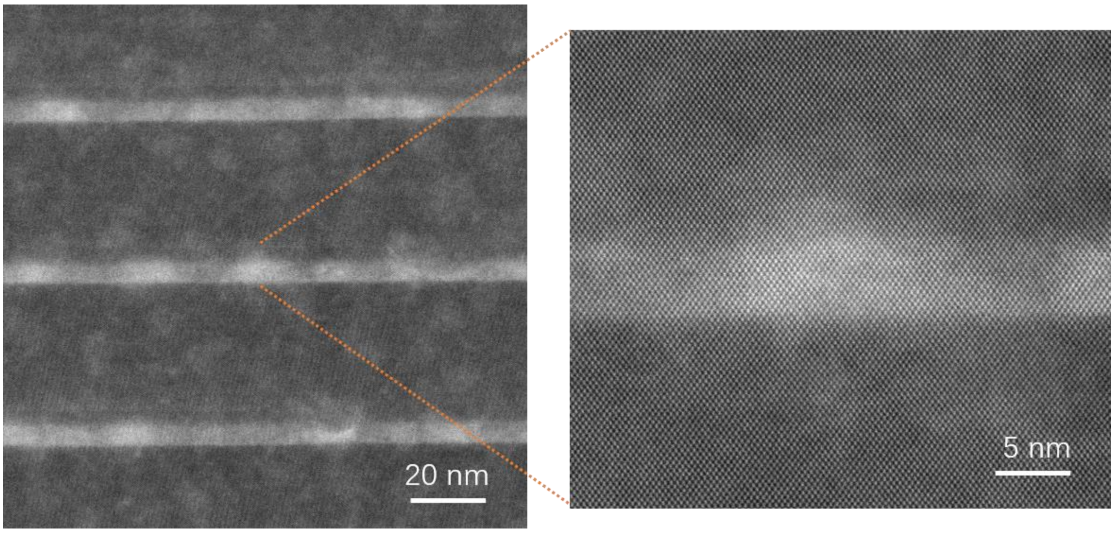

Figure S2. The high-resolution cross-sectional TEM images of the three-stack InAs/GaAs QD layers, together with a magnified view of a single QD.

Section II. Device Fabrication.

The fabrication flow is schematically shown in **Fig. S3**. It starts with deposition of ~200 nm SiO_2 on the as-grown wafer by inductively coupled plasma chemical vapor deposition (ICP-CVD) as a hard mask for PhC dry etching. Then a 400 nm ARP6200 electron beam resist is spin-coated on the surface of the SiO_2. The PhC pattern is defined using electron beam lithography (EBL) in the ARP6200 (**Fig. S3a**). The PhC pattern is then transferred from ARP6200 to the SiO_2 hard mask using reactive ion etching (RIE). Afterwards, the electron beam resist is removed using RIE with O_2 plasma. Inductively coupled plasma RIE (ICP-RIE) is performed subsequently to form the air-holes through the active region and the sacrificial layer (**Fig. S3b**). The residual SiO_2 hard mask is then removed by RIE.

The etched sample is transferred onto a glass substrate with Norland Optical Adhesive 61 (NOA61, an ultraviolet curing adhesive) and then left in a vacuum for an hour to remove microbubbles, followed by

five-hour exposure to ultraviolet for curing (**Fig. S3c**).

Citric- and HF-acids are used to selectively remove the GaAs substrate and the $Al_{0.8}Ga_{0.2}As$ sacrificial layer: the GaAs substrate is firstly thinned to a thickness of about 50 μm by grinding and then removed using critic acid. The $Al_{0.8}Ga_{0.2}As$ sacrificial layer is subsequently removed in a diluted hydrofluoric acid (**Fig. S3d**). The sample is again transferred onto a glass substrate with the same process of **Fig. S3c** to restore the up-down mirror symmetry, required by the off-Γ BICs.

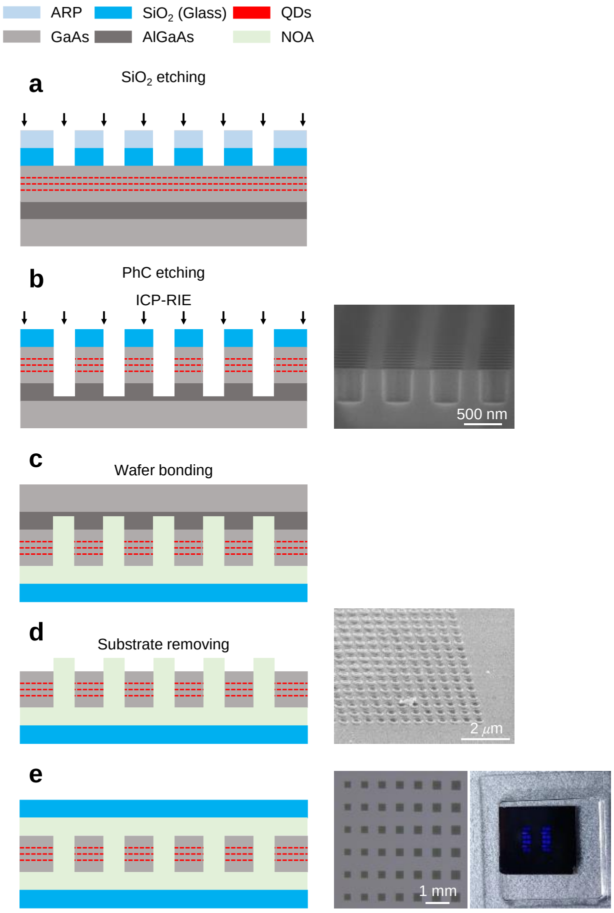

Figure S3. Device Fabrication. Schematic illustrations (the left column) and SEM images (or optical micrograph) of the fabrication procedure for mini-BIC lasers (the right column). **a.** PhC pattern defining using electron beam lithography (EBL) and reactive ion etching (RIE etching). **b.** Inductively coupled plasma RIE (ICP-RIE) dry etching to form the air-holes through the active region and the sacrificial layer. **c.** The sample is bonded to a transparent quartz substrate with NOA61 via an ultraviolent curing process. **d.** Selectively remove the GaAs substrate and the $Al_{0.8}Ga_{0.2}As$ sacrificial layer by citric- and HF-acids. **e.** Transfer again to ensure the mirror-flip symmetry.

Section III. The measurements of pumping spot size.

The pumping spot size is measured by microscope graticules. As shown in **Fig. S4**, the optical micrographs of the pumping spot together with microscope graticules are used to estimate the spot sizes. The outlines of microscope graticules are periodic curves, whose period is 10 μm. By fitting the outlines of microscope graticules with sinusoidal function, the size of a single pixel under 50x objective can be calibrated as 0.128 μm pixel^{-1}. Knowing the spot size in the pixel coordinate system, the actual diameter of spot thus can be obtained as 5.4 μm.

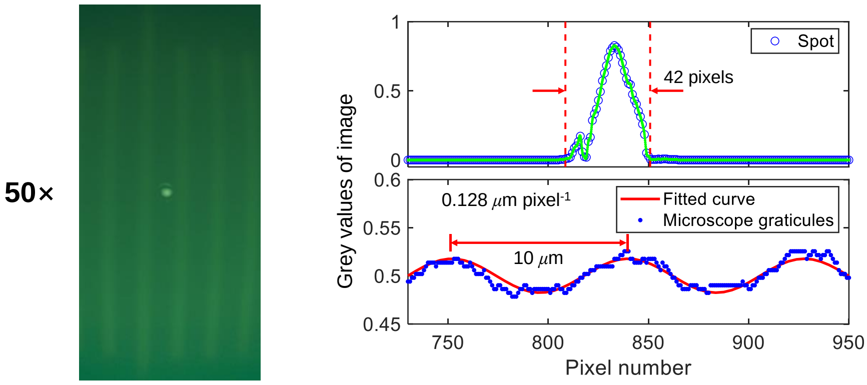

Figure S4. The optical micrograph of the spot with microscope graticule under 50x objective (the left column) and the according grey values from image used to estimate the spot size (the right column).

Section IV. Mode simulation for the mini-BIC lasers with *a* = 495 nm.

The accidental BIC essentially results from the destructive interference of radiative waves. In photonic crystal with infinite thickness, the coupling between bands of different Γ orders is forbidden because of orthogonality. However, in the case of photonic crystal slabs, the orthogonality is broken, and interband coupling is allowed via the continuum coupling within near fields, the destructive interference of which induces BICs[1]. Unlike the at-Γ BICs, the off-Γ accidental BICs can be continuously adjusted by parameter tuning. As long as the mirror-flip and inversion symmetry are kept, tunable accidental BICs can be found[1, 2]. **Fig. S5** depicts the fundamental TE band of bulk PhC slabs with *a* = 495 nm and corresponding Q factor (black lines), where off-Γ accidental BICs can be found. M_{11} mode of the mini-BIC with N_a = 13 is close to one of the accidental BICs.

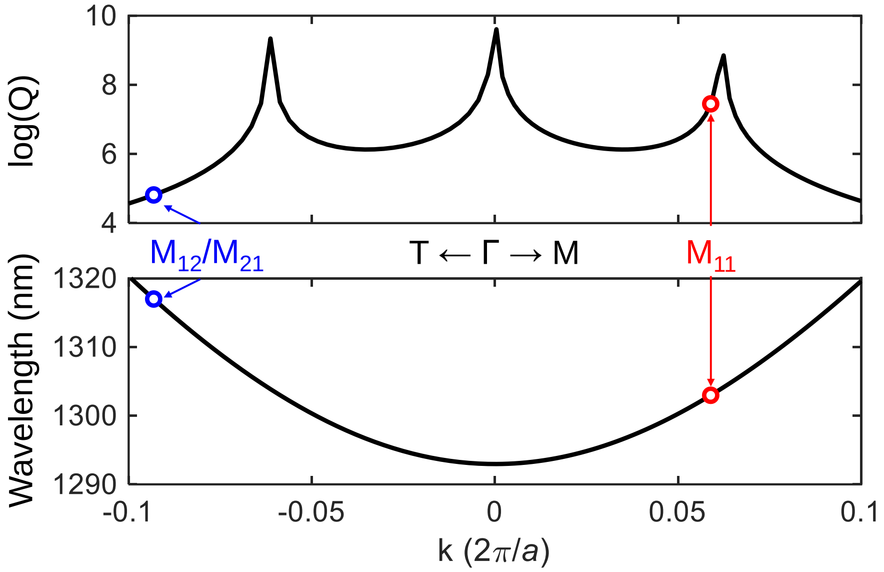

Figure S5. The wavelength and Q factor as a function of wave vector are shown in the lower and upper panel. For the mini-BIC laser with N_a = 13, the wavelengths of M_{11} and M_{12}/M_{21} are 1302 nm and 1316 nm, and the according Q factors are 4.1×10^7 and 6.6×10^4, respectively.

Section V. The effect of region B in mini-BIC.

To reveal the mirror effect of photonic bandgap (PBG) region (region B) in experiment, we fabricate several samples without region B and with varied N_a and *a*. PL spectra of PhC cavities (region A) without

region B are demonstrated in **Fig. S6a**. A series of mode peaks can be observed in the cavities with N_a = 50, which originates from the momentum quantization in confined PhC structures. The increase of constant lattice *a* gives rise to the redshift of resonant wavelengths (the left panel of **Fig. S6a**). With cavity size (N_a) decreasing, the resonant wavelengths redshift and meanwhile the wavelength intervals increase (the right panel of **Fig. S6a**), resulting from the larger spacing δk (equal to π/L, L is the size of cavity) between quantized modes. However, due to the larger $k_{//}$ of cavity modes, the smaller cavity with N_a = 15 suffers more from the light leakage so that do not exhibit any resonant cavity modes.

Furthermore, we compare the PL spectra of PhC cavity (only Region A), PBG region (only Region B) and mini-BIC cavity (containing Region A and B). As shown in **Fig. S6b**, no obvious resonance can be observed in PhC cavity with only region A. In the structure with only PBG region, Fabry-Perot (FP)-like modes can be found at 1270 nm, 1292 nm and 1314 nm, with a spacing of 22 nm, slightly different from the wavelength spacing in the FP cavity, which is $\lambda^2/2nL$ = 29 nm (λ = 1270 nm, n = 3.4 and $L = N_a \cdot a + b$ = 8.0 μm). In the mini-BIC cavity, the Q factors of modes are dramatically enhanced, indicating that the lateral leakage of light is effectively suppressed by the PBG boundary around the PhC cavity.

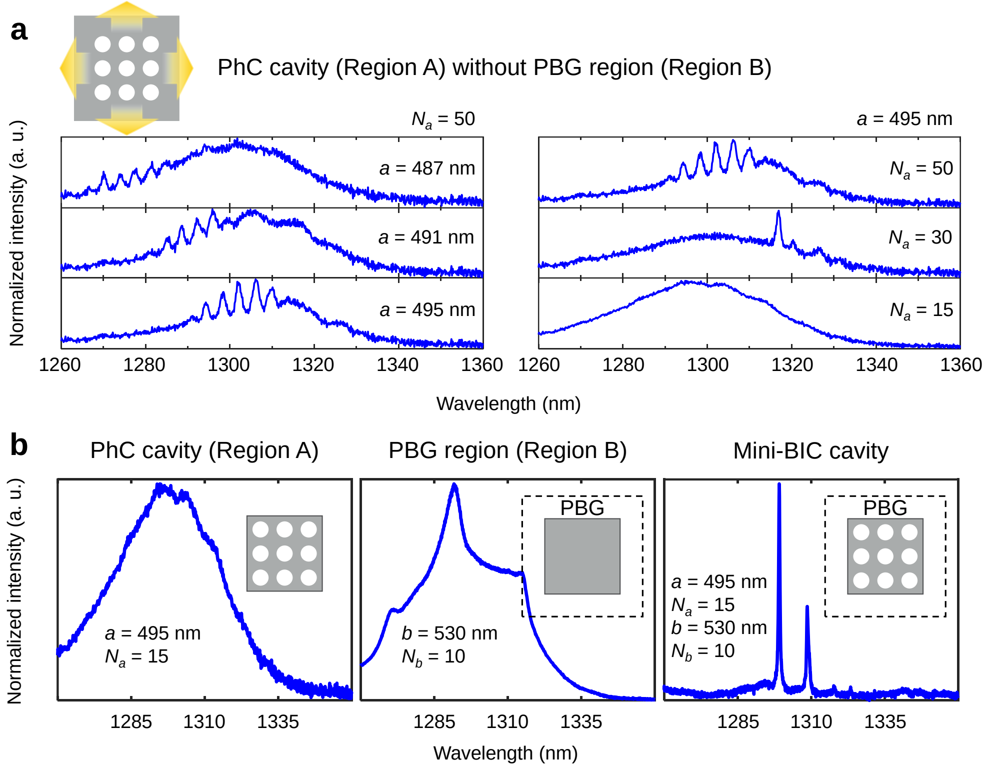

Figure S6. a. PL spectra of PhC cavities (region A) without photonic bandgap (PBG) region (Region B). The left panel shows the results from cavities with a fixing N_a = 50 and a varied *a* of 487 nm, 491 nm and 495 nm, while the right panel is the results from cavities with a fixing *a* = 495 nm and a varied N_a of 50, 30 and 15. **b.** The comparison of PL spectra of PhC cavity (only Region A), PBG region (only Region B) and mini-BIC cavity (containing Region A and B), indicating that the lateral leakage of light is effectively suppressed by the PBG boundary around the PhC cavity.

Section VI. Calculated spontaneous emission factor (β).

The soft turn-on shoulder near the threshold (**Fig. 2c**) allows the extraction of the spontaneous emission factor (β) of our device, which is determined mostly by the shape of the light in-light out (L-L) curve below and near threshold. To evaluate the β of the fabricated mini-BIC laser, the experimental L–

L plot is compared with theoretical curves calculated by using rate equations of microcavity semiconductor lasers[3-5]. Carrier density (N) and photon density (P) in the cavity are described by the following rate equation model:

$$\frac{dN}{dt} = \eta\frac{P_{in}}{\hbar\omega_p V} - \frac{N}{\tau_{sp}} - - \frac{N}{\tau_{nr}} - v_g g(N)P \qquad (1)$$

$$\frac{dP}{dt} = -\Gamma v_g g(N)P + \Gamma\frac{\beta N}{\tau_{sp}} - \frac{P}{\tau_p} \qquad (2)$$

where η is the absorption ratio of the pump laser in the active region, ω_p is the frequency of the pump laser, V is the active volume, v_g is the group velocity, Γ is the confinement factor, τ_{sp} is the spontaneous emission lifetime, τ_{nr} is the non-radiative recombination lifetime, and τ_p is the cavity photon lifetime. A linear gain model $g(N) = g_0(N-N_0)$ is assumed, where N_0 is the transparency carrier density of the gain material. The best fit to the measured data is obtained with an estimated β value of 0.13. This β value is considerably comparable with previously reported from the QD PhC lasers[5] and attributed to the effective photon localization by the mini-BIC cavity.

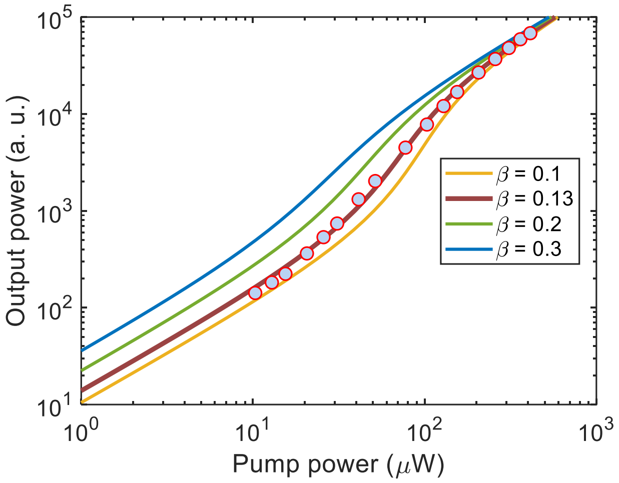

Figure S7. Corresponding logarithmic L–L plot of the min-BIC laser with N_a = 13 and a = 495 nm. Dots show the experimental data and the solid lines are theoretically calculated results for various values of β by using rate equation analyses.

Section VII. Second-order coherence of the emitted light in our device.

Corresponding measurements of the second-order autocorrelation function ($g^{(2)}(\tau)$) were carried out using a Hanbury-Brown and Twiss configuration. In our case, the coherence time of emission is shorter than the timing resolution of the detectors, so that it is difficult to observe $g^{(2)}(0) = 2$ in the thermal regime with very low emission rates. Thus the value of $g^{(2)}(\tau)$ as a function of pumping power is measured under pulsed excitation. In details, the devices are optically excited by a 780 nm pulse laser (10 ps, 86 MHz) through a microscope objective with a numerical aperture (NA) of 0.65. The signal emitted from the sample is collected by the same objective, and then routed into a single-mode fiber. A grating filter was used to filter the signal to be measured. Then the filtered signal was sent to a 50:50 fiber beam splitter (BS) and two superconducting nanowire single-photon detectors (SNSPD) for intensity autocorrelation measurement. Herein, the low coupling efficiency of our device to the fiber impose constraint on measuring $g^{(2)}(\tau)$ in the thermal regime at low emission rates.

The result shows a clear transition from thermal emission ($g^{(2)}(0)$ = 1.2 at 20 μW, below threshold) to

coherent state ($g^{(2)}(0)$~1 at 30 μW, above threshold), serving as a reliable indicator for lasing (**Fig. S8a**). We find that $g^{(2)}(0)$ value decays at a pumping power lower than 18μW instead of approaching the theoretical value $g^{(2)}(0) = 2$ for an ideal thermal light source. That is because the coherence time of spontaneous emission drops below the detection limit of our set-up, which has been reported and discussed in previous work[6, 7]. The measured histograms at pumping power of 15 μW, 20 μW, 25 μW and 30 μW are presented in **Fig. S8b** respectively.

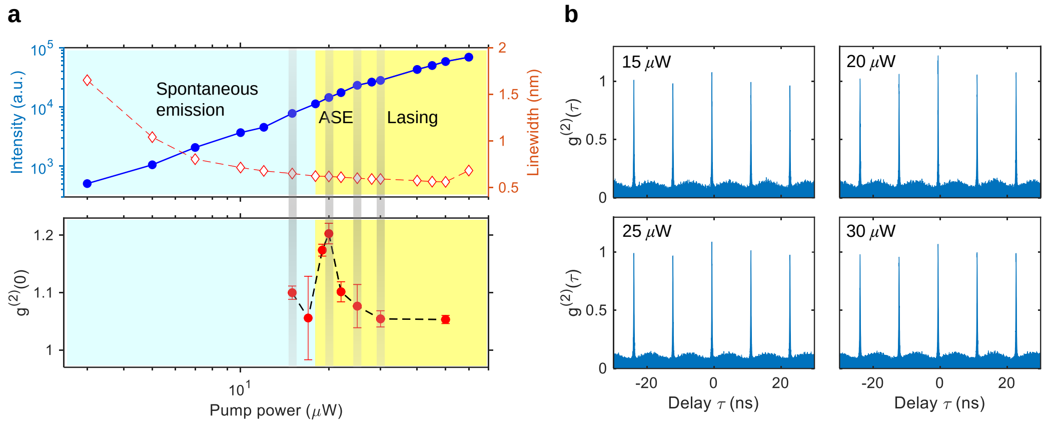

Figure S8. Second-order photon correlation function measurement. **a.** Integrated output intensity and linewidth of the mode M_{11} as a function of the pump power shows a threshold behavior around 18 μW (top panel). Second-order correlation functions at zero delay time $g^{(2)}(0)$ as a function of the excitation power exhibits $g^{(2)}(0)$ approaching unity above a pumping power of 20 μW, indicating that coherent lasing has been achieved (bottom panel). A clear transition from amplified spontaneous emission (ASE, a thermal state with $g^{(2)}(0) > 1$) to stimulated emission (a coherent state with $g^{(2)}(0) \sim 1$) can be observed. The error bars of $g^{(2)}(0)$ correspond to the standard errors originating from variations of the correlation function intensity for neighboring pulses. **b.** The measured histograms at different pumping power marked with shaded areas in **a**.

Section VIII. Resonant wavelengths in samples with the lattice constant of 495 nm and different N_a.

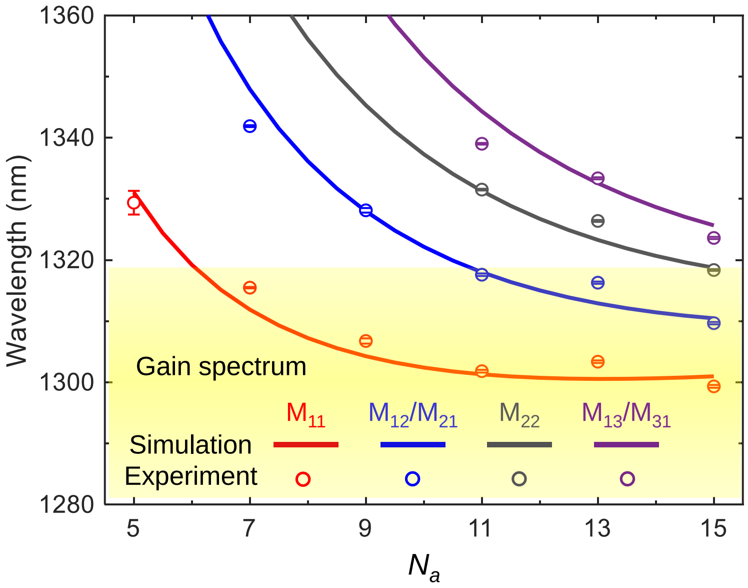

Figure S9. Measured resonant wavelengths of four modes (circles with error bars) in samples with the lattice

constant of 495 nm and different N_a show good agreements with simulation results (solid lines). The yellow shaded region shows the range of gain spectrum.

Section IX. Single-mode lasing spectra for the mini-BIC lasers.

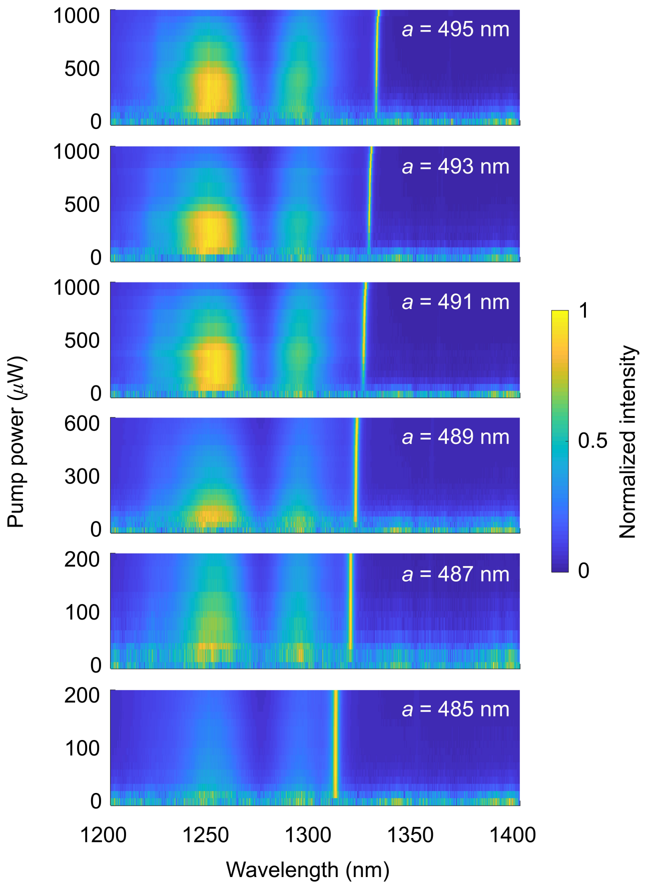

Figure S10. The lasing spectra of the BIC lasers with N_a fixed at 5 and lattice constant a varying from 485 nm to 495 nm. The emissions at ~1250 and ~1300 nm correspond to the luminescence from the excited and ground states of the QD.

Section X. The discussion on material absorption in mini-BIC lasers.

The simulated Q factor of mini-BIC modes is predicted to be extremely high, more than 10^7 in the optimum cases. Yet the difference between theoretical and experimental Q factor is over 3 orders of magnitude. Such differences were also observed in other reported BIC lasers as well. On the one hand, defects resulting from the fabrication imperfections and scattering losses at the heterostructure interface due to momentum mismatch are not considered in the simulation. On the other hand, the simulation object is built as passive structures without any loss, whose refractive index has only the real part. Therefore, the simulated Q factor would be much closer to the measured Q factor of passive cavity but rather different from the active cavity of lasers with the complex refractive index. Rather high Q factors above 10^5 or even 10^6 have been achieved in passive BIC[8] or mini-BIC cavity[9]. However, most of lasers based on BIC cavity has the Q factor of only several thousand[10-18].

The difference may be mainly caused by the photon absorption by the active medium around the laser cavity as all localized modes would penetrate, partly, into the boundary region. When lasing action happens, the material gain in the cavity is positive, while outside the cavity where pumping intensity falls

off, the material gain can be smaller or even negative. Taking mini-BIC cavity for example, its reflective boundary (region B) can be viewed as a lateral DBR structure, and thus the mode fields of mini-BIC cavity would extend several periods into region B outside the central cavity. Therefore, channels of photon loss can also form around the cavity. The degradation of Q factor brought by material absorption has been demonstrated in experiment by Ref. 18, where the measured Q factor of Si_3N_4 before and after the transfer of active 2D materials WS_2 is about 5000 and 2500. Similar results can also be found in Ref. 19.

Section XI. The discussion on the parameters of photonic bandgap boundary.

The photonic bandgap boundary is decided by two parameters, periodicity b and size N_b.

To explore how period b influences the mini-BIC mode, we fabricate and test a series of structures with a = 495 nm and b varying from 510 nm to 560 nm. As is shown in **Fig. S11**, the change of period b makes little impact on the resonant wavelength. However, the quality factor generally declines with b increasing and drops markedly when b is larger than 535 nm (**Fig. S11a**). The decline in Q may be the result of the joint effect by the change of bandgap as well as the severe scattering by the gap energy Δ between regions A and B. With the periodicity b increasing from 510 to 560 nm, M_{11} moves firstly to the center and then the edge of bandgap (**Fig. S11b**). Besides gap energy Δ monotonously increases (details in Ref. 9), which excites many scattered momentums (**Fig. S11c**). High reflection at the center of bandgap would be helpful for good lateral light trapping, while severe scattering induced by larger gap energy would reduce the Q factor. For a device with a = 495 nm and N_a = 13, we found an optimal design at b = 530 nm with a relatively high Q of 2561.

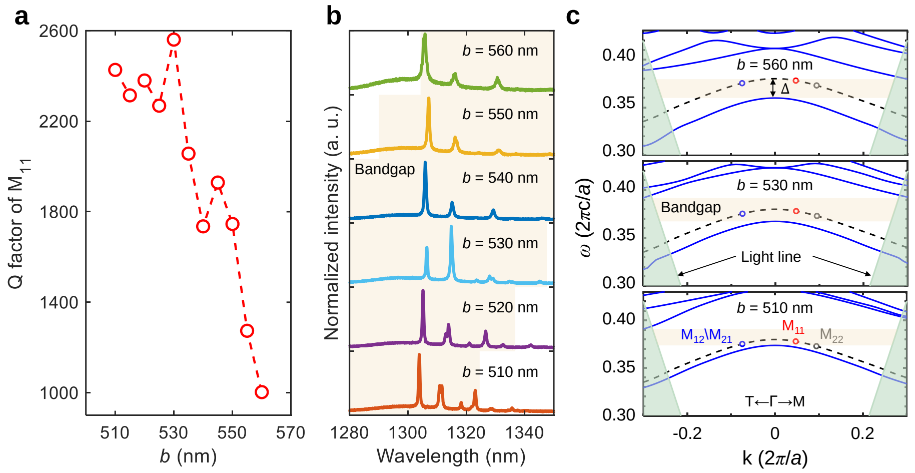

Figure S11. The influence of period *b* on the mini-BIC structure. a. The Q factor of M_{11} mode with b varying from 510 nm to 560 nm. **b.** Normalized spectra under varying periodicity b from 510 nm to 560 nm. The yellow region shows the spectral region of photonic bandgap. **c.** The calculated bulk bands of region A (black dashed line) and region B (blue) under the periodicity b of 510 nm, 530 nm and 560 nm. M_{11}, M_{12}/M_{12} and M_{22} are represented by red, blue and green circles respectively. The shaded green region is the light cone. Gap energy Δ monotonously increases with b varying from 510 nm to 560 nm.

Besides, N_a and N_b are two factors that affect lateral leakage. With the larger $k_{//}$ of the mode, the smaller cavity with smaller N_a suffers more from the lateral leakage loss, which can be significantly suppressed

by proper design of reflective boundary. In the mini-BIC structure, N_a directly controls the size of resonant modes (therefore resonant wavelengths and mode intervals), as is shown in **Fig. S9**. Indeed, lateral leakage can be directly controlled by N_b. For a given b, the gap energy Δ between TE A and TE B is set, lateral leakage loss can be effectively suppressed in an exponential manner of $e^{-\Delta \cdot L_b}$ by increasing boundary thickness L_b ($L_b = N_b \cdot b$), while the cost is a larger PhC footprint[8].

In our physical picture about mini-BIC cavity structure, best lateral confinement is achieved when the mode is in the high-reflection band of region B with little scattering at the interface between region A and B. It is meant that the mode should be designed in the bandgap of region B with a proper gap energy (Too large gap would induce severe scattering, while too small gap is insufficient to provide effective confinement.). Therefore, the optimal suppression of lateral leakage is the result of combined adjustment of all parameters of a, N_a, b and N_b. The resonances of modes are mainly decided by a and N_a. The energy band (bandgap and gap energy Δ) of region B is mainly controlled by b. The boundary thickness L_b is mainly decided by N_b. Considering the exponential manner ($e^{-\Delta \cdot L_b}$) of lateral leakage suppression by increasing boundary thickness L_b, the Q factor of confined mode would dramatically increase and then gradually tend to a maximum with N_b continuously increasing.

Reference

1. Yang, Y., et al., *Analytical Perspective for Bound States in the Continuum in Photonic Crystal Slabs.* Physical Review Letters, 2014. **113**(3): p. 037401.
2. Hsu, C.W., et al., *Observation of trapped light within the radiation continuum.* Nature, 2013. **499**(7457): p. 188-191.
3. Bjork, G. and Y. Yamamoto, *Analysis of semiconductor microcavity lasers using rate equations.* IEEE Journal of Quantum Electronics, 1991. **27**(11): p. 2386-2396.
4. Nomura, M., et al., *Ultra-low threshold photonic crystal nanocavity laser.* Physica E: Low-dimensional Systems and Nanostructures, 2008. **40**(6): p. 1800-1803.
5. Zhou, T., et al., *Continuous-wave quantum dot photonic crystal lasers grown on on-axis Si (001).* Nature Communications, 2020. **11**(1): p. 977.
6. Pan, S. H., et al., *Dynamic hysteresis in a coherent high-β nanolaser*. Optica, 2016. **3**(11): p. 1260–1265.
7. Badolato, A., et al., *Self-tuned quantum dot gain in photonic crystal lasers*. Physical Review Letters, 2006. **96**(12): p. 127404.
8. Jin, J., et al., *Topologically enabled ultrahigh-Q guided resonances robust to out-of-plane scattering.* Nature, 2019. **574**(7779): p. 501-504.
9. Chen, Z., et al., *Observation of miniaturized bound states in the continuum with ultra-high quality factors.* Science Bulletin, 2022. **67**(4): p. 359-366.
10. Contractor, R., et al. *Integrable and steerable vortex lasers using bound states in the continuum*. in *Frontiers in Optics + Laser Science APS/DLS*. 2019. Washington, DC: Optica Publishing Group.
11. Ha, S.T., et al., *Directional lasing in resonant semiconductor nanoantenna arrays.* Nature Nanotechnology, 2018. **13**(11): p. 1042-1047.
12. Huang, C., et al., *Ultrafast control of vortex microlasers.* 2020. **367**(6481): p. 1018-1021.
13. Kodigala, A., et al., *Lasing action from photonic bound states in continuum.* Nature, 2017. **541**(7636): p. 196-199.
14. Mohamed, S., et al. *Topological charge engineering in lasing bound states in continuum*. 2020. arXiv:2012.15642.
15. Wu, M., et al., *Bound State in the Continuum in Nanoantenna-Coupled Slab Waveguide Enables Low-Threshold Quantum-Dot Lasing.* Nano Letters, 2021. **21**(22): p. 9754-9760.
16. Wu, M., et al., *Room-Temperature Lasing in Colloidal Nanoplatelets via Mie-Resonant Bound States in the Continuum.* Nano Letters, 2020. **20**(8): p. 6005-6011.

17. Wang, Y., et al., *Highly Controllable Etchless Perovskite Microlasers Based on Bound States in the Continuum.* ACS Nano, 2021. **15**(4): p. 7386-7391.

18. Ge, X., et al., *Laterally confined photonic crystal surface emitting laser incorporating monolayer tungsten disulfide.* npj 2D Materials and Applications, 2019. **3**(1): p. 16.

19. Wu, S., et al., *Monolayer semiconductor nanocavity lasers with ultralow thresholds.* Nature, 2015. **520**(7545): p. 69-72.
